# Spatial frequency sensitivity in macaque midbrain

**DOI:** 10.1038/s41467-018-05302-5

**Published:** 2018-07-20

**Authors:** Chih-Yang Chen, Lukas Sonnenberg, Simone Weller, Thede Witschel, Ziad M. Hafed

**Affiliations:** 1 Werner Reichardt Centre for Integrative Neuroscience, Tuebingen University, 72076 Tuebingen, BW Germany; 2 Graduate School of Neural and Behavioural Sciences, International Max Planck Research School, Tuebingen University, 72074 Tuebingen, BW Germany; 3 Hertie Institute for Clinical Brain Research, Tuebingen University, 72076 Tuebingen, BW Germany; 4 Master’s Program for Neurobiology, Tuebingen University, 72076 Tuebingen, BW Germany

## Abstract

Visual brain areas exhibit tuning characteristics well suited for image statistics present in our natural environment. However, visual sensation is an active process, and if there are any brain areas that ought to be particularly in tune with natural scene statistics, it would be sensory-motor areas critical for guiding behavior. Here we found that the rhesus macaque superior colliculus, a structure instrumental for rapid visual exploration with saccades, detects low spatial frequencies, which are the most prevalent in natural scenes, much more rapidly than high spatial frequencies. Importantly, this accelerated detection happens independently of whether a neuron is more or less sensitive to low spatial frequencies to begin with. At the population level, the superior colliculus additionally over-represents low spatial frequencies in neural response sensitivity, even at near-foveal eccentricities. Thus, the superior colliculus possesses both temporal and response gain mechanisms for efficient gaze realignment in low-spatial-frequency-dominated natural environments.

## Abstract (web summary)

In primates, the superior colliculus (SC) contributes to rapid visual exploration with saccades. Here the authors show that the superior colliculus preferentially represents low spatial frequencies, which are the most prevalent in natural scenes.

## Introduction

The primate superior colliculus (SC) is a visual-motor structure important for transforming visual signals into behaviorally appropriate gaze shift commands^1–5^. Even though much is known about the primate SC’s afferent and efferent connections, as well as its physiological visual and eye-movement-related neural response characteristics, such knowledge has predominantly been obtained using highly impoverished stimuli, like small spots of light presented over an otherwise uniform background. However, ecological constraints^6–8^ on both visual perception and eye movements imply that the primate SC, like other brain regions, should best function if its neurons’ properties were well matched with the properties of the environment.

Among such properties is the preponderance of low spatial frequencies in natural scene statistics^9,10^. In early visual areas of the primate, such preponderance is well matched with a variety of observations, including coarse-to-fine neural image analysis^11–14^ (also ref. ^15^ in cats) and neural image filtering kernels that are suitable for natural scene statistics^16–18^. Curiously, such observations are often also used to account for motor rather than perceptual effects, for example on manual and saccadic reaction times (RTs)^19–21^, even though these early visual areas may be viewed as being more relevant for perception rather than action.

In this study, we hypothesized that the primate SC’s importance in guiding action^1,2,5,22^ should make it as well matched to spatial properties present in natural scenes as early visual areas, and in a manner that is highly conducive of behavioral motor effects with eye movements. We specifically tested the ability of rhesus macaque SC neurons to detect low spatial frequency visual stimuli. We found that these neurons do so much earlier than for high spatial frequencies, and independently of neural sensitivity to a given spatial frequency. Moreover, we found that at the population level, macaque SC neural sensitivity to spatial frequency was primarily low-pass in nature, meaning that both SC response time and SC response strength are particularly efficient when visually analyzing the low spatial frequencies that are abundantly present in natural scenes. These observations have allowed us to predict, with high fidelity, our animals’ saccadic RT patterns as a function of spatial frequency based solely on SC visual response strength and latency measurements obtained from completely different experimental sessions not involving saccadic responses. We believe that our findings clarify important visual functions of the primate SC^7^, complementary to this structure’s more well-studied motor^2^ and cognitive^5,23^ functions.

## Results

### Faster SC responses to low spatial frequencies

**Fig. 1:**
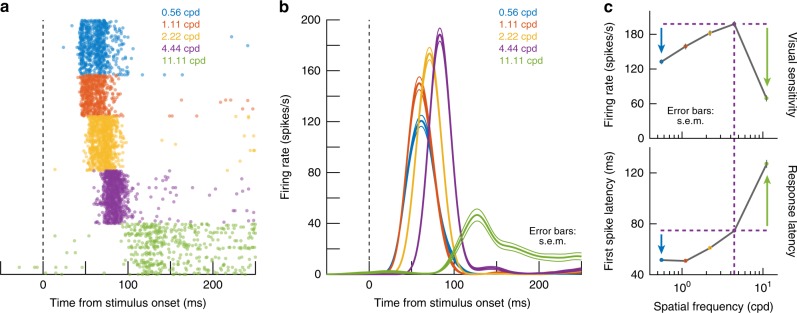
Rapid primate superior colliculus (SC) detection of low spatial frequencies. **a** Visual responses of an example SC neuron to different spatial frequencies. Raster plots show times of individual action potentials with different trials from a given spatial frequency stacked in rows. Different spatial frequencies presented are color-coded in the figure, and they are only grouped here for easier visualization; spatial frequencies were randomly interleaved in the experiment itself. There was a rank ordering of response latency by the same neuron as a function of spatial frequency, with the lowest spatial frequencies (e.g. 0.56 and 1.11 cpd) evoking the shortest-latency neural responses. **b** The same neuron emitted the strongest visual responses for 4.44 cpd even though these strong responses came significantly later than for lower spatial frequencies. Thus, response latency and sensitivity were dissociated. **c** The top panel plots peak stimulus-evoked firing rate as a function of spatial frequency for the same neuron. Visual responses were strongest for 4.44 cpd. On the other hand, in the lower panel, latency to first visually evoked spike at 4.44 cpd was longer than for lower spatial frequencies but shorter than for higher ones (e.g. colored arrows). Thus, there was a dissociation between neuronal response sensitivity and response latency. Error bars in **b**, **c**, when visible, denote s.e.m

We recorded visual responses in passively fixating macaque monkeys^24,25^. During fixation, we presented a high contrast, static sine wave grating filling the visual response field (RF) of a neuron (Methods). We sized the grating manually in every session in order to fill as much of a given neuron’s RF as possible, based on RF measurements with a spot of light^7,25^, while at the same time ensuring that the grating did not extend out into suppressive RF surrounds (Methods). The spot-based measurements revealed RF areas in our recorded population that were in the range of 0.3–303 deg^2^ (mean: 70.8 deg^2^; median: 28.24 deg^2^), and these areas exhibited, on average, a monotonic increase with neuronal preferred eccentricity. We randomly varied the spatial frequency of the grating that we presented to a given neuron from trial to trial, and we noticed a systematic rank ordering of neural response latencies as a function of spatial frequency. For example, in the example neuron of Fig. 1a, visually evoked action potentials arrived earliest for gratings of 0.56 or 1.11 cycles deg^−1^ (cpd), and their latency progressively increased for higher spatial frequencies. This is reminiscent of coarse-to-fine image coding properties of early visual areas^11–15^, but it still violated an expected inverse relationship between response latency and sensitivity previously reported in the SC^26^. Visual sensitivity in this neuron was highest for 4.44 cpd (i.e. highest peak firing rate in Fig. 1b), but response latency at this spatial frequency was significantly longer than at lower frequencies (first-spike latency at 4.44 cpd: 74.61 ± 0.42 ms s.e.m.; first-spike latency at 0.56 cpd: 51.49 ± 0.82 ms s.e.m.; *p* = 1.14 × 10^−38^, Ranksum test). This meant that plotting either visual sensitivity (Fig. 1c, top) or latency (Fig. 1c, bottom) as a function of spatial frequency revealed a significant dissociation: the preferred spatial frequency in terms of response sensitivity (i.e. peak firing rate) was 4.44 cpd, whereas the preferred spatial frequency in terms of response latency was much lower. Note that we measured peak, rather than average, response within a suitable time window after stimulus onset (Methods) exactly to ensure that the sensitivity tuning curve in Fig. 1c (top) was immune to the different neural response latencies associated with different spatial frequencies in Fig. 1c (bottom).

**Fig. 2:**
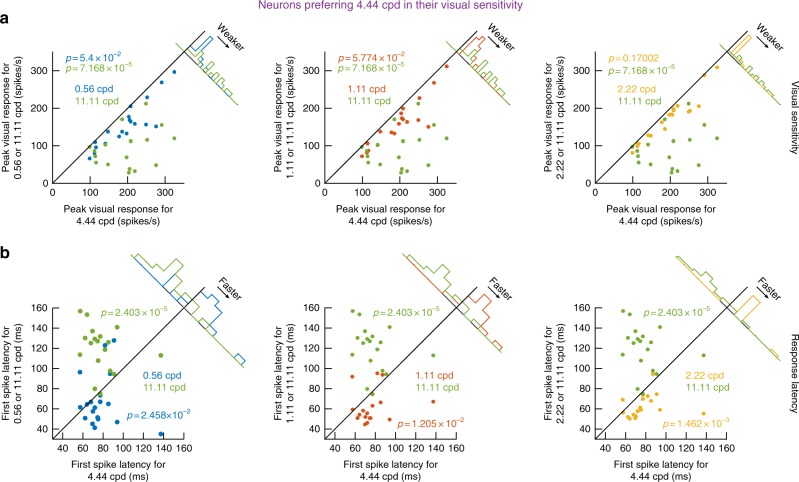
Rapid primate SC detection of low spatial frequencies independent of neural sensitivity. **a** For neurons showing the highest visual responses to 4.44 cpd gratings (*N* = 19), we plotted in each panel visual response strength for either higher or lower spatial frequencies (*y*-axes) against response strength for 4.44 cpd. As expected, response strength was always highest for 4.44 cpd. *p*-values are indicated in each panel, reflecting a comparison between either the higher or lower spatial frequency (color-coded according to the legend) to 4.44 cpd using a Ranksum test. **b** First-spike latency for 4.44 cpd gratings (*x*-axes) and either lower or higher spatial frequencies (*y*-axes). Even though 4.44 cpd gratings always evoked the strongest response (**a**), first-spike latency was either longer or shorter than the latency for other gratings (*p*-values are indicated in each panel); whether first-spike latency for the preferred spatial frequency (4.44 cpd) was longer or shorter simply depended on the rank-ordering of spike timing observed in Fig. 1. Thus, early SC visual sensation for low spatial frequencies is independent of response strength

We confirmed the dissociation between visual sensitivity and visual latency across our recorded population. To illustrate this here in the clearest possible fashion, we first demonstrate the basic effect by summarizing data from an example preferred spatial frequency, and we then present all the preferred spatial frequencies that we sampled in our experiments. For the example preferred spatial frequency, consider, say, neurons preferring 4.44 cpd in terms of visual sensitivity (i.e. their peak stimulus-evoked firing rate was the highest for 4.44 cpd gratings). For these neurons, we plotted either such sensitivity (Fig. 2a) or instead response latency (Fig. 2b) for different spatial frequencies; in all cases, we compared responses to those obtained when the preferred 4.44 cpd gratings were presented. For example, in the leftmost panel of Fig. 2a, we plotted response sensitivity to 0.56 cpd (bluish dots) or 11.11 cpd (greenish dots) as a function of response sensitivity to 4.44 cpd. Since the neurons preferred 4.44 cpd, response sensitivity was naturally lower for both 0.56 cpd and 11.11 cpd (*p*-values for statistical tests are shown in the figure). Similar results were obtained in the middle and rightmost panels of Fig. 2a for other spatial frequencies. Thus, in terms of visual sensitivity (Fig. 2a), all presented spatial frequencies other than 4.44 cpd expectedly elicited weaker neural responses than 4.44 cpd, since all the neurons selected in this analysis preferred 4.44 cpd. However, despite such preference, visual response latency (Fig. 2b) in the same neurons was either significantly shorter or significantly longer than the latency observed for 4.44 cpd (*p*-values for statistical tests are shown in the figure), and following a very simple rule: for 0.56, 1.11, and 2.22 cpd spatial frequencies, response latencies were shorter than for 4.44 cpd, whereas they were longer for 11.11 cpd. Again, for all of these spatial frequencies, response sensitivity was weaker than for 4.44 cpd. Thus, faster SC detection of low spatial frequencies occurs independently of visual sensitivity to a given spatial frequency.

**Fig. 3:**
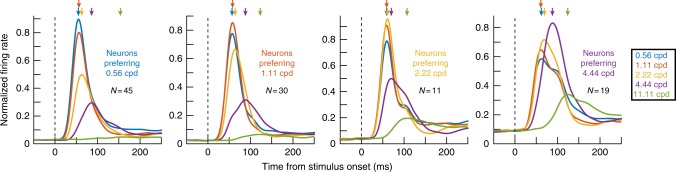
Dissociation between primate SC response strength and latency as a function of spatial frequency. The effect in Fig. 2 persisted for neurons preferring other spatial frequencies. In each panel, we took only neurons preferring one spatial frequency out of the five that we presented in the experiment (e.g. 0.56 cpd in the leftmost panel and 4.44 cpd in the rightmost panel, and so on for the other panels). As expected, gratings of non-preferred spatial frequencies expectedly evoked weaker visual responses than the preferred spatial frequency in each panel (e.g. 0.56 cpd had the highest firing rate in the leftmost panel, and 4.44 cpd had the highest firing rate in the rightmost panel, and so on for the other panels). However, regardless of visual sensitivity to a given spatial frequency, the rank ordering of visual burst times as a function of spatial frequency was similar across all panels (indicated here schematically by the downward arrows highlighting the time of peak visual response for each spatial frequency). For example, responses to 2.22 and 4.44 cpd gratings always came later than responses to 0.56 and 1.11 cpd gratings regardless of which spatial frequency the neurons preferred. Note that we did not have enough neurons preferring 11.11 cpd to include in this analysis (see Figs. 6 and 7 for reasons why). The numbers of neurons contributing to each panel are indicated in the figure

This observation also persisted when we considered neurons preferring other spatial frequencies. In Fig. 3, we plotted visually evoked responses for different spatial frequencies, but after separating neurons in each panel according to their preferred spatial frequency. The leftmost panel shows neurons responding the strongest for 0.56 cpd, and the rightmost panel shows neurons responding the strongest for 4.44 cpd, and so on for other panels. Yet, and as can be seen from the arrows indicating the times of peak visual responses for each spatial frequency, the lowest two spatial frequencies always evoked the fastest responses followed by a systematic increase in response latency with increasing spatial frequency; again, this happened regardless of neural preference for spatial frequency.

**Fig. 4:**
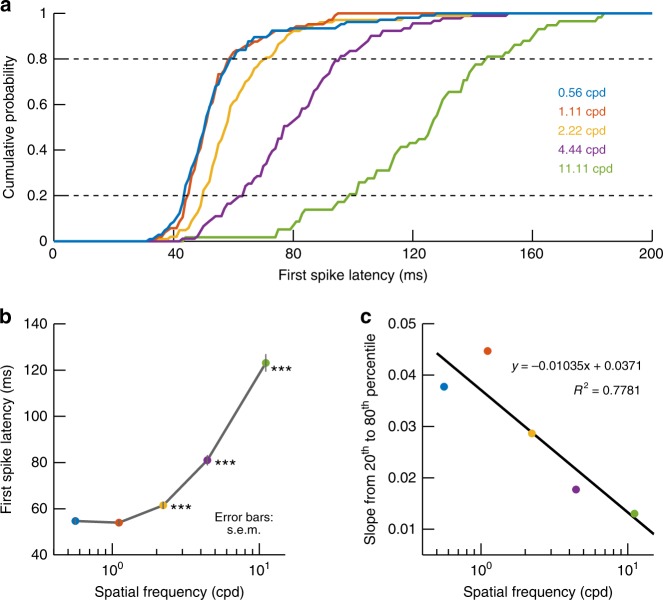
Early visual sensation for low spatial frequencies by the primate SC. **a** Cumulative histograms of first-spike latency (Methods) across neurons, separated by spatial frequency. For each neuron, we measured the average first-spike latency of the evoked visual response after a given spatial frequency grating was presented on multiple trials. We then repeated the measurement for other spatial frequencies. The evoked response consistently came earlier for low spatial frequencies than for high spatial frequencies. **b** The rank-ordering of spatial frequencies in **a** is also seen when plotting mean first-spike latency across all neurons as a function of spatial frequency. Low spatial frequencies evoked a visual response earlier than high spatial frequencies. Error bars denote s.e.m. across neurons, and asterisks indicate *p* < 0.001 when comparing first-spike latency for 0.56 cpd to that in each of the other spatial frequencies. **c** Variability of first-spike latency was higher for higher spatial frequencies. We plotted the slope of the cumulative histograms in **a** between the 20th and 80th data percentiles as a function of spatial frequency. This slope progressively decreased, suggesting a progressive increase in first-spike latency variability across neurons with higher spatial frequencies

We also further analyzed response timing properties of our SC neurons. We plotted cumulative histograms of first-spike latencies across neurons (Methods). The lowest two spatial frequencies (0.56 and 1.11 cpd) consistently evoked the shortest visual response latencies followed by a monotonic increase with increasing spatial frequency (Fig. 4a, b, including statistical tests), and such increase was also associated with greater response time variability (Fig. 4c). Moreover, this increase in primate SC visual response latency as a function of spatial frequency persisted for either purely visual or visual-motor SC neurons (i.e. both superficial and intermediate-layer SC neurons; Supplementary Fig. 1), and it was also independent of differences in response latency between upper and lower visual field primate SC representations^7^ (Supplementary Fig. 2a, b). Thus, even though upper visual field neurons tended to have smaller RF sizes and higher spatial frequency preferences^7^, the rank ordering of visual response latencies as a function of spatial frequency persisted. We also confirmed that primate SC visual response latencies after the presentation of classic, small spots of light were longer than with 0.56 and 1.11 cpd gratings, since spots are broad-spectrum stimuli that also include high spatial frequency components (the spots were also much smaller than the gratings, especially for extra-foveal neurons; Supplementary Fig. 2c).

**Fig. 5:**
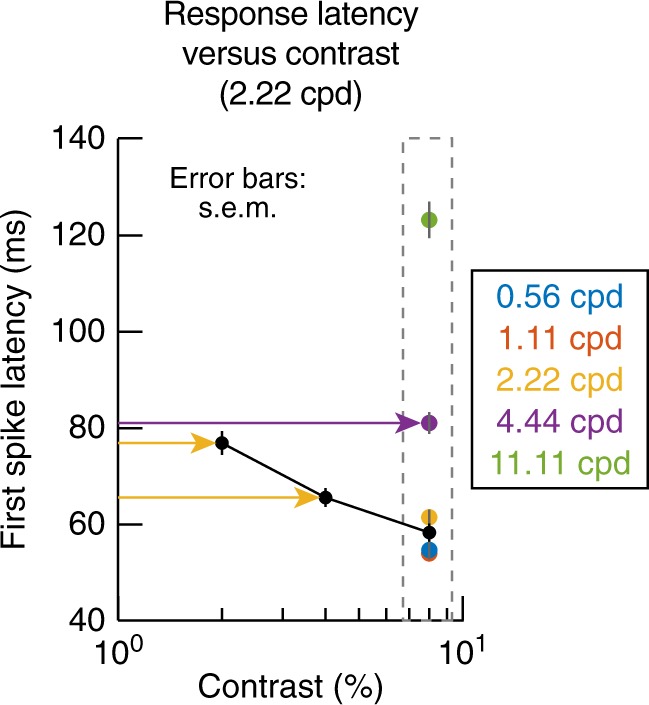
Relationship between stimulus contrast and first-spike latency in SC visual responses. For a population of neurons recorded from the same animals (Methods), we tested contrast sensitivity at 2.22 cpd, and we analyzed first-spike latency as a function of grating contrast (black curve). Since lower contrasts were associated with lower visually evoked firing rates^25^, this also resulted in longer first-spike latencies than with 80% contrast gratings (black curve). However, despite this, even these longer latencies were still shorter than the first-spike latencies observed for 4.44 cpd gratings with 80% contrast (the colored dots surrounded by a dashed gray box show the same first-spike latency data from Fig. 4, testing the effect of spatial frequency on first-spike latency at 80% grating contrast; latencies for 4.44 cpd were still longer than latencies for 2.22 cpd, even when the latter had only 20% contrast). Thus, even with only 20% or 40% contrast, first-spike latency for 2.22 cpd was still shorter than first-spike latency for 4.44 cpd at 80% contrast. This means that the increased first-spike latencies that we observed at 4.44 cpd (with high-contrast gratings) were not necessarily due to reduced sensitivity for such gratings. The great majority of neurons used in this figure had preferred eccentricities <15 deg (Fig. 6c)

Finally, we wondered whether longer visual response latencies for high spatial frequencies might have occurred because of lower real or perceived contrast at these frequencies. For example, factors like the optical modulation transfer function of the eyeball^27^ and retinotopic eccentricity^28^ might limit contrast sensitivity for high spatial frequencies; in turn, lower contrast sensitivity would be expected to be associated with lower visual response strengths in SC neurons^25,26,29^. Might it then be the case that a longer visual response latency at, say, 4.44 cpd than at 0.56 cpd is a simple result of reduced sensitivity for 4.44 cpd gratings? We think that this is unlikely. For example, a high-frequency fall-off in the contrast sensitivity function is expected to occur for frequencies larger than ~10 cpd in adult macaques^30^, whereas we noticed increased SC neural response latencies already at 2.22 and 4.44 cpd (and with high contrast stimuli; Figs. 1–4). Similarly, Figs. 1–3 indicate that a neuron could exhibit higher visual sensitivity for a higher spatial frequency but still possess a longer first-spike latency, suggesting that the longer latency was not a consequence of weaker sensitivity. We nonetheless investigated this issue further by analyzing first-spike latency from a population of neurons (*N* = 100) in which we fixed spatial frequency (at 2.22 cpd) and varied stimulus contrast (Methods). Figure 5 shows the results of these analyses. In this figure, the black curve shows first-spike latency for 2.22 cpd gratings as a function of grating contrast. As expected, lower stimulus contrast, associated with lower neural sensitivity^25,26,29^, resulted in higher first-spike latencies (Fig. 5; black curve). However, even at 20% contrast, first-spike latency for 2.22 cpd gratings was still shorter than first-spike latency for 4.44 cpd gratings at 80% contrast (the dashed box shows the same data as those summarized in Fig. 4 from our main experiment with 80% stimulus contrast to facilitate the comparison to the lower-contrast data; see the horizontal colored arrows). Thus, our results so far were not confounded by potential impacts of neural contrast sensitivity on visual response latencies.

### Over-representation of low spatial frequency sensitivity

**Fig. 6:**
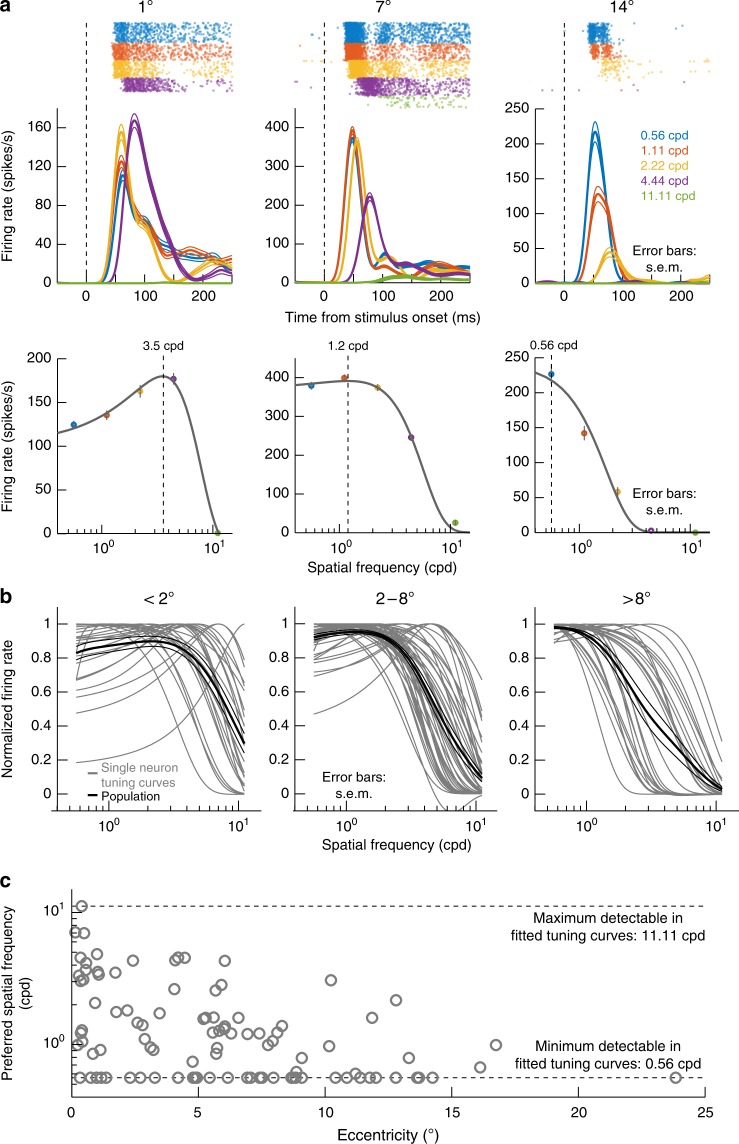
Predominantly low-pass spatial frequency sensitivity preferences in the primate SC. **a** Example visual responses of three SC neurons preferring different retinotopic eccentricities (1, 7, or 14 deg). Each panel in the top row plots firing rate as a function of time from stimulus onset for gratings presented within each neuron’s visual RF; color codes indicate spatial frequency, and firing rate curves show mean and s.e.m. (across trials) as thick and thin lines, respectively. Raster plots above the firing rate curves show the times of individual action potentials across trials, as in Fig. 1a. The near-foveal neuron (1 deg) preferred higher spatial frequencies than the more eccentric neurons. The bottom panels show tuning curves for the same neurons (plotting peak visual response as a function of spatial frequency) using Eq. (1) in Methods. Dashed vertical lines indicate the neurons’ preferred spatial frequencies based on the tuning curves. **b** Tuning curves from all neurons in our population, grouped into three different eccentricity bins. Thin curves show individual tuning curves, and thick black curves show the mean curve within a given panel, along with s.e.m. error bars across neurons. Regardless of eccentricity, population tuning curves were primarily low-pass (thick black curves), and this effect got stronger for more eccentric neurons (compare panels). **c** Preferred spatial frequency as a function of neuronal preferred eccentricity. Near-foveal neurons had a broad range of preferred spatial frequencies, as might be expected, but there was still low spatial frequency preference. Preferred spatial frequency was selected in this figure as the peak in fitted tuning curves, like those shown in **a**. Thus, for extremely low-pass or high-pass neurons, the preferred spatial frequency indicated was only an estimate that was cut-off by the end of the fitted curves constrained by our sampled spatial frequencies (dashed horizontal lines)

Besides rapidly detecting low spatial frequencies, being able to efficiently guide rapid eye movement behavior implies that the primate SC’s pattern analysis machinery might also be more sensitive to such low spatial frequencies at the population level. Indeed, we found primarily low-pass characteristics in the population even at near-foveal eccentricities. Figure 6a shows sensitivity tuning curves of three example neurons from different retinotopic eccentricities, and Fig. 6b, c summarizes the population. For these analyses, we used a classic difference-of-Gaussians fit to estimate individual tuning curves, as is done in the literature (Methods)^11,31^. The range of preferred spatial frequencies was expectedly higher at near-foveal eccentricities than at extra-foveal ones (Fig. 6c)^7^ (also consistent with cortical visual areas^32–35^), but the overall population curves were primarily low-pass (black curves in Fig. 6b). This is in strong contrast to primary visual cortex (V1), in which band-pass tuning was shown^32^ to be more prevalent at similar eccentricities to those in which we saw low-pass SC tuning. Also, and as stated above (e.g. Figure 5), the primarily low-pass nature of our SC tuning curves was not explained by perceptual contrast sensitivity curves, since adult macaque contrast sensitivity curves up to about 8 deg or more of eccentricity^30,36^ reveal higher sensitivity than would be predicted by our neuronal tuning curves alone. Therefore, our results from Fig. 6 suggest that the SC, unlike V1, over-represents low spatial frequencies in terms of visual sensitivity, in addition to its boosting of such spatial frequencies in terms of response latency (Figs. 1–5).

**Fig. 7:**
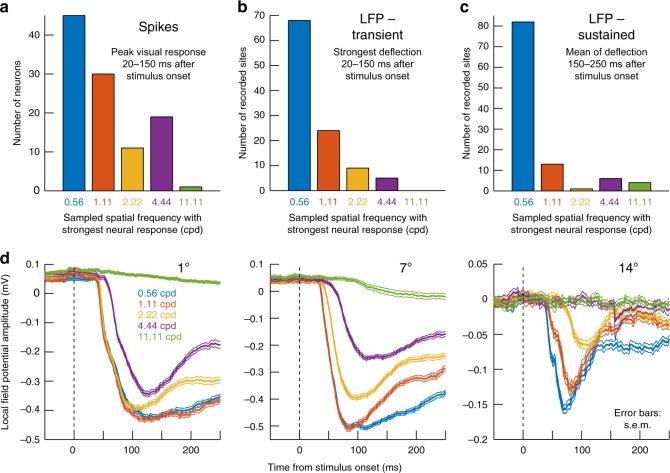
Low-pass primate SC spatial frequency filtering characteristics. **a** Distribution of preferred spatial frequencies in our population. In this analysis, we binned neurons according to the presented spatial frequency (out of five values) eliciting maximal response. More neurons were driven the strongest by the lowest spatial frequency (0.56 cpd). **b** We performed a similar analysis but on the transient evoked local field potential (LFP) response (Methods; also see **d** for example evoked LFP responses, which are negative going). The number of electrode penetrations showing maximal response for 0.56 cpd was even higher than for the isolated neurons in **a**. **c** This effect was even stronger in the sustained LFP response starting after 150 ms from stimulus onset. Thus, at the population level reflected by LFP signals, the primate SC is primarily tuned to low spatial frequencies. **d** Stimulus-evoked LFP responses from the same electrode penetrations in which the example neurons of Fig. 6a were isolated and recorded. In the LFP, all three electrode tracks, regardless of eccentricity, showed a preference for low spatial frequencies (stronger negative deflections), even in the near-foveal SC region where the neuron preferring 3.5 cpd in Fig. 6a was isolated. This means that the SC over-represents low spatial frequencies in neural sensitivity. Error bars denote s.e.m.

We explored the over-representation of low spatial frequencies further by first counting the number of neurons responding the most for 0.56 cpd as opposed to our other sampled spatial frequencies. These neurons accounted for 42% of our population, and no other single spatial frequency recruited as many neurons (Fig. 7a). Interestingly, this over-representation of low spatial frequencies became even more obvious when assessing local population activity reflected in field potentials (local field potential; LFPs), recorded simultaneously around our electrode tips along with the isolated neurons (Methods). We measured either the evoked (Fig. 7b) or sustained (Fig. 7c) local population activity after grating onset (Methods), and the great majority of our electrode locations (64% for the evoked response and 77% for the sustained response), whether in near-foveal or extra-foveal locations, picked up the strongest responses for the lowest spatial frequency (Fig. 7b, c). This effect can be better appreciated when inspecting raw LFP traces from the same three example electrode penetrations from which the three example neurons of Fig. 6a were isolated (Fig. 7d). Even though the near-foveal neuron in Fig. 6a (leftmost panel) responded the most for the 4.44 cpd grating, the local population around the electrode in the same experiment still showed the strongest stimulus-evoked deflection (as well as sustained response) for 0.56 and 1.11 cpd gratings (Fig. 7d, leftmost panel). In other words, at the population level, even near-foveal SC eccentricities over-represent low spatial frequencies. Similar effects were also observed for the other two example eccentricities in Fig. 7d. This dissociation between LFP responses and individual neuron tuning properties is additionally interesting because it confirms that the low-pass nature of tuning curves in Fig. 6 was not an artifact of the monkeys not being able to see higher-frequency gratings particularly well; if this were the case, then individual neuron tuning curves at foveal sites should have shown the same low-pass predominance observed in Fig. 7. Therefore, the SC over-represents low spatial frequencies both in terms of neural sensitivity (Figs. 6, 7) as well as response latency (Figs. 1–5).

### Faster scanning of low spatial frequencies with saccades

**Fig. 8:**
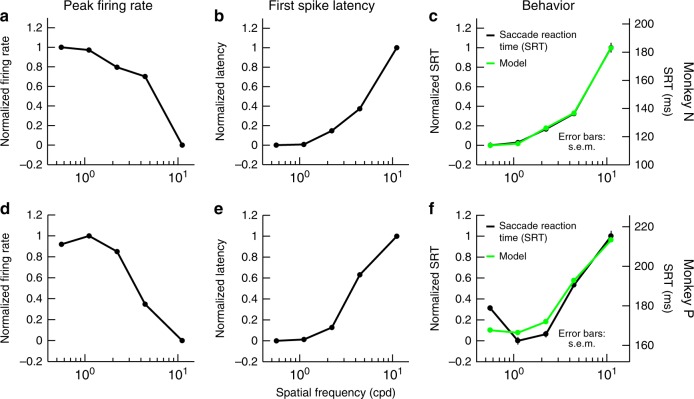
Strong correlation between SC visual response properties and saccadic reaction time (RT). **a** For monkey N, we plotted average response strength as a function of spatial frequency for all neurons covering an eccentricity similar to an eccentricity used in separate behavioral sessions requiring a saccade to the gratings (Methods). Firing rates were normalized in the range of 0–1 from lowest to highest response. Low spatial frequencies were associated with higher responses, as shown in Fig. 6. **b** We performed a similar analysis for neural response latency from the same neurons; this time, low spatial frequencies were associated with more rapid neural responses, as shown in Fig. 4. **c** The black curve shows the monkey’s saccadic RT as a function of spatial frequency from completely different behavioral sessions, in which an orienting saccade towards the grating was required as quickly as possible after the grating’s onset (Methods). The green curve shows a linear combination of the neural curves in **a**, **b** (Methods). There was a high correlation between SC visual response properties and saccadic RT, even though the monkey never made target-directed saccades to the gratings during the recordings of our neural data. **d**–**f** Similar analyses for monkey P. Error bars are defined in the figure where appropriate (note that the error bars for saccadic RT are sometimes too small to see; error bars for neural parameters were shown in earlier figures). For monkey N, model parameters *a*, *b*, and *c* from Eq. (2) in Methods were −0.458, 0.537, and 0.465, respectively, and the percentage explained variance of the data by the model was 99.9%. For monkey P, the parameter values were −0.427, 0.463, and 0.5, respectively, and the percentage explained variance of the data by the model was 89.7%. In both animals, there was a roughly equal correlation between either response strength or first-spike latency and saccadic RT, because the first two parameters in Eq. (2) had roughly equal absolute values

Finally, we related SC visual response latency and sensitivity to behavior. In separate sessions, our monkeys generated visually guided saccades to gratings of different spatial frequencies^37^ instead of simply maintaining fixation as in our recording sessions. Saccadic RT expectedly increased with increasing spatial frequency^20,37^, and our goal here was to ask how such RT increase was correlated with SC visual response properties (even when these visual responses were recorded in the complete absence of saccadic orienting to the gratings, and in different experimental sessions). For each monkey, we plotted average peak firing rate as a function of spatial frequency for neurons with RFs near the location of the saccade target used in the behavioral experiments (Methods), and we confirmed the low-pass nature of SC visual sensitivity in each animal individually (Fig. 8a, d showing peak firing rate as a function of spatial frequency). Similarly, we plotted first-spike latency as a function of spatial frequency for the same neurons and observed earlier responses for low spatial frequencies (Fig. 8b, e). Simple linear correlations of visual response strength (Fig. 8a, d) and latency (Fig. 8b, e) with RT (Fig. 8c, f; black curves) in a given monkey were high and allowed predicting the monkey’s behavior remarkably well (Fig. 8c, f; green curves). Moreover, there was a roughly equal correlation between either visual response sensitivity or first-spike latency and RT in each monkey, as revealed by the parameter values of the coefficients in Eq. (2) of Methods when obtaining the green curves of Fig. 8c, f. Thus, SC visual response properties (both strength and latency) are very strongly correlated with how saccades depend on spatial frequency. These results are in line with our recent findings that even fine-scale changes in SC visual sensitivity due to, say, microsaccades remain highly correlated with fine-scale changes in RT^37,38^. Finally, we also related these observations to human performance and showed that saccade efficiency in a visual search task is also strongly spatial-frequency dependent, even when ensuring high visibility of gratings with relatively high spatial frequencies (Supplementary Fig. 3).

## Discussion

We found preferential SC representation of low spatial frequencies in terms of both response latency and strength. We believe that these results place the SC in an ideal position to facilitate orienting in natural environments, which are dominated by low image spatial frequencies^9,10^. Consistent with this, it was found that saccadic RTs are significantly faster in natural scenes, after ensuring matched stimulus visibility^21^, although these authors did not suggest a potential role for the SC in these observations. We also performed a visual search task with high target visibility (Supplementary Fig. 3) and found strong dependence of saccadic timing on spatial frequency. In this regard, our analysis of monkey saccadic RTs (Fig. 8) is particularly intriguing, because it further suggests that the SC may indeed be instrumental (along with other visual areas) in facilitating these human observations. Specifically, we were able to account for each animal’s RT patterns with high fidelity based solely on SC visual responses collected from different experimental sessions and during passive fixation. In other words, SC visual response properties were sufficient to estimate whether saccadic RTs were going to be fast or slow. Even in monkey P, for which RT at the lowest spatial frequency deviated slightly from our model (Fig. 8f), the increase in RT at the lowest spatial frequency was associated with a decrease in visual response strength at the same frequency in this animal (Fig. 8d), further suggesting that both visual response properties (strength and latency) can account for RT variability. We believe that these results make sense in hindsight. If one were to expect any visual brain areas to be optimized for natural scene spatial-frequency statistics, it should be those areas that have privileged access to the motor output, like the SC.

Our results are also interesting because they highlight the importance of the primate SC’s visual functions. Historically, this structure was studied heavily from the perspective of motor control^2^, with more recent interest focusing on cognitive processes related to attention, decision making, and target selection^5,23^. However, the primate SC is also a visual structure, and it is the primary visual structure in lower animals. Indeed, recent results have examined visual properties of the primate SC, for example to color stimuli, more closely^8,39–41^. We particularly think that our results may provide a possible mechanism for allowing the primate SC to preferentially process face-like stimuli, as was also recently observed^42^, and to mediate known preferential orienting patterns to such stimuli^43^. Of course, one cannot directly equate spatial frequency processing to face processing, because factors like face size (i.e. distance from the observer) would affect the range of spatial frequencies that are present in a given facial image. However, for a given operating range of potential face distances that an animal might encounter in its everyday life, it is conceivable that rapid visual detection of faces and other ecologically relevant objects may be a worthwhile endeavor for a structure like the SC that can mediate rapid orienting behaviors.

Moreover, the primate SC’s visual functions could potentially contribute to certain phenomena associated with blindsight^44^. In this condition, patients with V1 loss conscious perception but nonetheless exhibit residual sensory, cognitive, and motor capabilities that may be mediated by V1-bypassing pathways. Some primate models of blindsight point to a possible SC role in guiding saccades under this condition^45,46^. Also, studies of spatial frequency sensitivity of human blindsight patients show spatial frequency cutoffs near ~4 cpd^47–49^, similar to the capabilities of our SC neurons (Fig. 6b). In addition, there have been careful experiments performed in which inactivating lateral geniculate nucleus (LGN) and/or V1 resulted in loss of SC visual responses only in intermediate layers, but not in the retino-recipient superficial layers^50^, suggesting that the (superficial) SC indeed has access to visual signals from the retina (in addition also to signals from extra-striate cortical areas). Of course, all of this does not necessarily imply that other visual pathways bypassing V1 (for example, geniculo-cortical pathways) are not the most critical pathways for blindsight^51^. Rather, it merely suggests that the SC’s role in visually guided behavior, whether in normal or pathological conditions, is more nuanced than suggested by classic oculomotor studies employing simple spots of light.

Related to spatial frequency cutoffs, we found primarily low-pass SC tuning (Figs. 6, 7). Of course, at near-foveal eccentricities, where higher resolution vision dominates, band-pass tuning curves were observed, but the overwhelming population result was low-pass, unlike in V1. In anesthetized marmosets, band-pass SC tuning was suggested^52^, although it is not clear how this may have depended on eccentricity in that study. These authors also observed low-pass tuning in some of their neurons like in our case. In any case, our observations of primarily low-pass tuning curves is reminiscent of LGN tuning curves^53^ rather than V1 ones, which tend to be band-pass at similar eccentricities to those in which we observed low-pass behavior^32^. However, such similarity to LGN tuning does not necessarily trivialize our results because the SC, while receiving both retinal and V1 projections, does not receive direct projections from the LGN. Also, unlike the retina and V1 (and potentially also LGN), the SC exhibits significantly higher spatial frequency preferences in the upper visual field when compared to the lower visual field^7^, suggesting that spatial vision by the primate SC is more sophisticated than being simply a trivial outcome of retinal and/or V1 projections.

Another potential point of difference between the SC and LGN/V1 might relate to neural effects related to peri-saccadic perceptual phenomena. Specifically, it was shown perceptually that around the time of saccades, low spatial frequency stimuli are suppressed more strongly than high spatial frequency stimuli^54^. These results have suggested that there may be selective suppression of the magnocellular pathway around the time of saccades, but neural evidence for this was not supportive^55–59^. Interestingly, when we recently tested for a correlate of this perceptual phenomenon in the SC, we indeed found selective suppression of low spatial frequencies, but only in intermediate-layer visual-motor neurons^37^. Thus, the presence of spatial frequency tuning in the primate SC is not only relevant for scene analysis as we suggest in this study, but it is also relevant during active perception states in which the visual system alters its response properties to alleviate consequences of spurious image signals coming from the retina when the eye moves. Interestingly, the difference between SC saccadic suppression and LGN/V1 saccadic suppression also suggests that evidence of magnocellular input from LGN and V1 to the SC^50^ does not necessarily imply that the SC inherits all of its visual processing capabilities from these structures. Rather, a distinct computation is performed when these inputs are wired to the SC, resulting in functionally distinct contributions of the different structures to perception and action. This holds true even though overall SC contrast sensitivity curves (e.g. Fig. 6b) might resemble, qualitatively, the shape of the contrast sensitivity curve of the magnocellular pathway in general^60^. Indeed, it may be the case that the SC, having access to a significant variety of visual inputs from the retina and beyond, integrates all such information in a manner that results in overall tuning characteristics that are most similar to the overall perceptual contrast sensitivity curve of the organism (when compared to other individual visual brain areas like LGN and V1). This similarity of the SC’s overall tuning characteristics to perceptual sensitivity characteristics allows the SC to mediate behaviors suitable for the natural environment, not only in the case of saccadic suppression as just described, but also in normal visual scene analysis that can be later used to support orienting responses.

In terms of response latencies, the dissociation between visual sensitivity and latency that we observed (Figs. 1–3, 5) is particularly noteworthy. It is normally accepted that SC visual response latency is inversely proportional to response strength^26^, and similar observations have also been made in V1^61^. However, we found that it is possible to have both stronger and later visual responses (e.g. Figs. 1–3, 5). This is a property of the apparently coarse-to-fine SC processing dynamic that we uncovered. Of course, it may be the case that dissociations between response strength and latency also exist in other early visual areas. For example, a subset of V1 neurons in one study exhibited this property^14^. Unfortunately, other studies of coarse-to-fine processing in early visual areas have relied on anesthetized monkeys and cats^11–13,15^, and have also not explicitly described a dissociation between visual sensitivity and latency. This complicates quantitative comparisons between our results and those in other early visual areas. For example, it would be interesting to know, with similar spatial frequency conditions as ours, how primate V1 response latencies at the population level can correlate with saccadic RT (as done in ref. ^62^ for stimulus contrast). It would also be interesting to know whether such correlation would be as high as for SC visual responses in our data (e.g. Fig. 8), as well as in our earlier results in which RT was modulated in a highly similar manner to SC visual responses by the occurrence of microsaccades^37,38^. Such comparisons can help clarify the importance of functional specializations of different brain areas when trying to understand and interpret the brain basis of behavior in primates^6^.

We also found that the SC can exhibit extremely short visual response latencies, especially for low spatial frequencies. This is in line with an SC role in guiding rapid orienting responses and with the SC receiving direct retinal projections^63^. This is also consistent with the fact that eye movements, including microsaccades, can be reflexively altered by visual stimuli with latencies earlier than the latencies of most higher-level cortical visual areas^64–66^.

In all, we believe that our results demonstrate that spatial vision capabilities of the primate SC are specifically organized to facilitate exploring natural scenes with rapid gaze shifts.

## Methods

### Ethics approvals

Monkey experiments were approved by regional governmental offices in Tuebingen. For the human experiments, ethics committees at Tuebingen University reviewed and approved our protocols. All human subjects provided written informed consent in accordance with the Declaration of Helsinki.

### Animal preparation

Monkeys P and N (male, *Macaca mulatta*, aged 7 years and weighing 7 and 8 kg, respectively) were obtained from the German Primate Center (Göttingen, Germany) and prepared for behavior and SC recordings earlier^24,25^. Briefly, we placed a recording chamber centered on the midline, and we angled it to point towards a stereotaxically defined point 1 mm posterior of and 15 mm above the inter-aural line. The chamber angle was posterior of vertical (by 38 and 35 deg for monkeys P and N, respectively).

### Monkey recording task

The monkeys sat in a primate chair placed in a dim room in front of a cathode ray tube (CRT) monitor having a refresh rate of 120 Hz. The monitor’s luminance profile was linearized using Gamma-correction, and we used eight-bit resolution for control of individual luminances (i.e. 256 levels of gray). The display was configured for a resolution of >22 pixels per deg, and the luminance of the standard gray background over which stimuli were presented was 21 cd m^−2^. Viewing distance was 45 cm, and we used a custom-built real-time experimental control system communicating with a dedicated computer running Psychophysics Toolbox^67–69^ for display control. This toolbox, run through Matlab (MathWorks, Inc.), allowed access to graphics card hardware, such that we could control displayed stimuli on a frame-by-frame basis. Additional details of our real-time experimental controller that was communicating with Matlab were provided in ref. ^24^.

The monkeys performed a pure fixation task while we recorded the activity of visually responsive SC neurons, as described in detail before^25,37^. Briefly, in each trial, we displayed a white fixation spot (8.5 × 8.5 min arc) over a gray background. Fixation spot luminance was 72 cd m^−2^, and it consisted of a 3 × 3 pixel white stimulus with the middle pixel of the array left at background contrast to aid in stabilizing gaze fixation^24^. After an initial fixation interval (400–550 ms), the fixation spot transiently dimmed for ~50 ms to reset microsaccadic rhythms^65,66^ and to also attract attention to the spot rather than to the RF stimulus. After an additional 110–320 ms, a stationary, vertical Gabor patch with 80% relative contrast (defined as *L*_max_ − *L*_min_/*L*_max_ + *L*_min)_ appeared for 300 ms within the neuron’s RF. The RF was estimated earlier in the session using standard saccade tasks involving onsets of spots of light at different spatial locations^7,25^, and the Gabor patch size was chosen for each neuron individually in order to fill as much of the RF as possible. We avoided increasing the patch size beyond the classical RF of a given neuron in order to avoid encroaching on suppressive RF surrounds. The spatial frequency of the patch, in cycles deg^−1^ (cpd), was varied randomly across trials (from among 0.56, 1.11, 2.22, 4.44, and 11.11 cpd). Grating phase was randomized from trial to trial, and the monkey was rewarded only for maintaining fixation; no orienting to the grating or any other behavioral response was required. We used only vertical gratings, but we confirmed that they elicit robust responses in the SC. In pilot data, we also confirmed that any potential orientation tuning in the SC was broad and included robust responses to vertical gratings^25,70,71^. For the most foveal neurons in our sample (e.g. see Fig. 6c), we found that RF shape was skewed outwards from the center of gaze, such that there were more locations farther in eccentricity from the RF hotspot that would stimulate a given neuron than locations nearer in eccentricity than the RF hotspot. This property, consistent with log-polar transformations in neural tissue, allowed us to slightly displace the grating outward from the fixation spot for these neurons (in order to avoid overlapping the spot visual stimulus with the grating visual stimulus) while still being able to effectively drive visual responses from these neurons.

We recorded from 115 neurons (monkey N: 60; monkey P: 55) with preferred eccentricities up to 24 deg. We excluded trials with microsaccades occurring within ±100 ms from stimulus onset because such occurrence can alter neural activity. In fact, the trials with microsaccades near stimulus onset were analyzed recently, from the same set of neurons, to explore spatial-frequency dependence of saccadic suppression in the SC^37^. Our focus here was to only analyze baseline visual activity and not activity modulated due to the presentation of peri-movement stimuli. We excluded 9 neurons from further analyses because they did not have >25 repetitions per tested spatial frequency after excluding the microsaccade trials. This number was our chosen threshold for the minimum number of observations in order to have sufficient confidence in our interpretations of the results. For the remaining 106 neurons that were included in the analyses, we collected >295 trials per neuron (average: 935 ± 271 s.d.).

In a subset of our analyses (e.g. Fig. 5), we compared neural activity in our neurons to activity (*N* = 100 neurons) recorded from the same two animals but under the condition in which spatial frequency was held constant at 2.22 cpd and grating contrast was varied from trial to trial (phase was also randomized as stated above). These contrast sensitivity data were described and analyzed in detail previously^25^, but the analyses presented in the current study (namely, first-spike latency; Fig. 5) were not described previously. 54/106 (50.9%) of the neurons recorded for the present study were also recorded in the same sessions as the contrast sensitivity manipulations in the previous study; thus, for these 54 neurons, each neuron was tested for both spatial frequency (the present study) and contrast sensitivity (the study of ref. ^25^).

### Monkey saccade RT task

In completely different purely behavioral sessions, we ran our monkeys on a simple saccade RT task, which we recently described in detail^37^. Briefly, the monkeys fixated, and a Gabor patch of 2 deg diameter could appear at 3.5 deg eccentricity either to the right or left of fixation. The patch was otherwise identical to that used in the recording task described above, and the fixation spot disappeared simultaneously with patch appearance in order to cue the monkeys to generate a targeting saccade towards the patch. We measured RT and correlated it with SC visual responses collected from completely different sessions and critically not involving a saccadic response at all (i.e. the recording task above). We analyzed 2522 trials from Monkey N and 3392 trials from Monkey P. As with the neural data above, we only analyzed trials without any microsaccades within 100 ms before or after Gabor patch onset, to avoid peri-movement effects on RT that were described in detail elsewhere from the same experimental sessions^37^.

### Human visual scanning task

Subjects sat in a dark room facing a computer display (CRT; 41 pixels per deg; 85 Hz), and head fixation was achieved through a custom-made chin/forehead rest. The display was linearized using Gamma-correction and had eight-bit grayscale resolution, and the standard gray background over which stimuli were presented had a luminance of 20.5 cd m^−2^. We collected data from eight subjects (three females and five males; five subjects were authors of the study).

Each trial started with an initial fixation spot presented at display center. After ~1030 ms of steady fixation, a search array consisting of 4 × 4 Gabor patches appeared. Each patch was 6.1 deg in diameter, and all 16 patches were distributed evenly in a grid layout across the display (the display spanned approximately ±17.1 deg horizontally and ±12.8 deg vertically). Grating contrast was set to maximum (100%), and all patches had the same spatial frequency within a given trial. Spatial frequency was altered randomly across trials from among six possible values (0.33, 0.66, 1.31, 1.97, 3.93, and 5.9 cpd). Moreover, all but one patch had the same orientation within a given trial (picked randomly across trials from all possible orientations with a resolution of 1 deg). The odd patch was tilted by 7 deg either clockwise or counter-clockwise from the orientation of all other patches, and the subjects’ task was to search for the oddly oriented patch and indicate whether it was tilted to the right or left from all other patches. The task was very difficult to perform during fixation (because of the small orientation difference in the oddball patch), and therefore required prolonged scanning of the entire grid array of patches with many saccades until the odd patch was found and correctly discriminated. This allowed us to obtain sufficient search performance data, with many inter-saccadic intervals that were the focus of our analysis (i.e. our goal was to investigate how inter-saccadic intervals were affected by spatial frequency). We collected 180 trials per subject (i.e. 30 trials per spatial frequency), but each trial had many more inter-saccadic intervals that could be analyzed (as detailed below). It is important to note here that for the spatial frequencies that we tested in this experiment, the contrast that we used (100%) was well above the threshold contrast of the human contrast sensitivity function^28,72^, including at extra-foveal eccentricities^28^. Thus, any differences in inter-saccadic intervals as a function of spatial frequency that we observed in our analyses were unlikely to be due to perceptual difficulties in seeing the higher spatial frequency patches that we tested.

### Neuron classification

We used similar neuron classification criteria to those used in our recent studies^7,25^. Briefly, a neuron was labeled as visual if its activity 0–200 ms after target onset in a delayed saccade task^7^ was higher than activity 0–200 ms before target onset (*p* < 0.05, paired *t*-test). The neuron was labeled as visual-motor if its pre-saccadic activity (−50 to 0 ms from saccade onset) was also elevated in the delayed saccade task relative to an earlier fixation interval (100–175 ms before saccade onset)^29^. Visual neurons were more superficially located across SC depth than visual-motor neurons as expected from this structure’s physiology and anatomy. Our results (e.g. spatial frequency rank ordering of response latencies) were similar for either visual or visual-motor neurons (except for small quantitative differences in visual response latency). As a result, we combined neuron types in analyses unless otherwise explicitly stated.

### Eye movement analyses

We measured eye movements in monkeys using scleral search coils, and we used a video-based eye tracker (EyeLink 1000, SR Research, Canada) for humans. We detected saccades and microsaccades using velocity and acceleration criteria detailed elsewhere^24^.

For the monkey recordings, we detected microsaccades in order to exclude trials with such movements occurring near stimulus onset (see above). For the monkey saccade RT task, we detected the targeting saccade after grating onset and measured its RT. We only considered trials in which there were no microsaccades within ±100 ms from target onset, because microsaccades near target onset alter RT^37,38^, and because these trials with peri-microsaccadic stimuli were analyzed separately elsewhere^37^.

For the human scanning task, we measured inter-saccadic intervals during search. The inter-saccadic interval was defined as the time period between the offset of one saccade and the onset of the next. We only considered saccades occurring between search array onset and trial end (i.e. button press) when computing inter-saccadic intervals. Moreover, we only analyzed trials in which there were no blinks during the entire period from which we were collecting inter-saccadic intervals. Because trials were long until subjects found the odd patch, meaning that we had many inter-saccadic intervals within any trial, removal of blink trials did not reduce our data set dramatically; in the end, we had a total of 3325–4743 accepted inter-saccadic intervals per spatial frequency in our analyses (from a total of 145–182 accepted trials per spatial frequency).

### Firing rate analyses

We analyzed SC visual burst strength by first converting spike times into firing rate estimates using convolution with a Gaussian kernel having 10 ms s.d. We then measured peak firing rate 20–150 ms after stimulus onset^37^. Note that we chose to look for peak firing rate as opposed to average firing rate exactly because different spatial frequencies were associated with different neural response latencies (e.g. Fig. 1); thus, we designed a liberal time window in which we could search for the peak visual response while still having estimates of neural sensitivity that were immune to differences in neural response latency.

We obtained spatial frequency tuning curves by plotting peak visual response as a function of grating spatial frequency^7^. Note that we did this on the raw data (i.e. we measured peak visual response for each tested spatial frequency). For one analysis (Fig. 6), we estimated tuning curves as difference-of-Gaussians functions. We performed a least-squares fit of the measurements at each tested spatial frequency to the following difference-of-Gaussians function:

1 $$f\left( x \right) = a_1 \ast e^{ - \left( {\frac{{x - b_1}}{{c_1}}} \right)^2} - a_2 \ast e^{ - \left( {\frac{{x - b_2}}{{c_2}}} \right)^2} + {B}$$

where *f* is firing rate, *x* is spatial frequency, *a*_1_ and *a*_2_ represent the amplitude of each Gaussian function, *b*_1_ and *b*_2_ represent the mean of each Gaussian function, *c*_1_ and *c*_2_ are the bandwidth of each Gaussian function, and *B* is the baseline firing rate (obtained from all trials as the mean firing rate in the interval 0–50 ms before Gabor patch onset). The goodness of fit was validated by computing the percentage of variance across stimuli accounted for by the model^73^. Only neurons that had >80% explained variance by the fit were included in summaries of tuning curve fits in Results (97 out of 106 neurons), but all neurons were included in all other analyses. We also note here that the tuning curves from the same neurons in this study were presented earlier in brief format to provide support for the conclusions of another independent study out of our laboratory^7^; however, the conclusions and analyses shown in the present study are novel and were not described elsewhere before.

We estimated the preferred spatial frequency of each neuron as the spatial frequency within the sampled range of 0.56–11.11 cpd for which the fitted tuning curve from the above equation peaked. To combine different neurons’ tuning curves (e.g. Fig. 6b), we first normalized the peak of the tuning curve of each neuron to 1. We then combined neurons and obtained a mean curve across neurons along with s.e.m. estimates.

We estimated first-spike latency using Poisson spike train analysis^74^. Most of our neurons had very little or no baseline activity, meaning that our estimate of first-spike latency using this method was very robust, and it gave us a sense of how quickly our neurons responded to the onset of a given stimulus. We computed population-level cumulative histograms of first-spike latency across neurons: for each neuron, we measured average first-spike latency of the evoked visual response when a given spatial frequency grating was presented on multiple trials, and we then sorted neurons according to their average first-spike latency.

We also performed first-spike latency analyses on the neurons from ref. ^25^ in order to explore the relationship between contrast sensitivity and first-spike latency (e.g. Fig. 5). The same procedure as described above was used, and we then plotted first-spike latency as a function of grating contrast.

### Local field potential analyses

We obtained local field potentials from wide-band neural signals using methods that we described recently^7,37^. We then aligned LFP traces on Gabor patch onset, and we measured evoked responses in two ways. First, we measured the strongest deflection occurring in the interval 20–150 ms after stimulus onset, to obtain a measure that we called the transient LFP response. Second, we measured the mean deflection in the period 150–250 ms after stimulus onset, to obtain what we referred to as the sustained LFP response. Since the LFP evoked response is negative going, when we refer to a peak LFP response, we mean the most negative value of the measured signal.

### Correlating saccade RTs to visual responses

Our approach was to ask whether RT from behavioral sessions can be related in a simple manner to visual response strength and first-spike latency from completely separate neural recording sessions in which no saccade to the patch was ever made. We used linear models of the form:

2 $${\mathrm {RT}}\left( {\mathrm {{PV,FSL}}},x \right) = {a} \ast {\mathrm {PV}}(x) + {b} \ast {\mathrm {FSL}}(x) + {c}$$

where *x* is spatial frequency, PV(*x*) is the average peak visual response of all included neurons for spatial frequency *x*; FSL(*x*) is the average first-spike latency of all included neurons for spatial frequency *x*; and *a*, *b*, *c* are model parameters. Since the behavioral RTs were experimentally obtained from horizontal targets at 3.5 deg eccentricity, we only included neurons with preferred RF locations centered within the range of 2–10 deg in eccentricity and ±45 deg in direction from horizontal (i.e. 46 neurons). Moreover, we separated each monkey’s neurons so that its own neural activity was used to predict its behavioral variability. Since we obtained similar conclusions when relating RT to either visual neurons alone or visual-motor neurons alone, we combined neuron types in the shown analyses to maximize the numbers of neurons used. We also normalized the range of RT values that we observed to the range from 0 to 1, with 0 corresponding to the shortest RT (e.g. that obtained from the lowest spatial frequency). We similarly normalized the range of peak visual response and first-spike latency. We then fit the best parameters to Eq. (2) above that matched the data. Because our data showed that RT was negatively correlated with PV and positively correlated with FSL, we imposed a constraint that parameter *a* had to stay in the range of −1 to 0, parameter *b* had to stay in the range of 0 to 1, and parameter *c* had to stay in the range of −0.5 to 0.5.

### Data availability

All data presented in this paper are stored in institute computers and are available upon reasonable request.

## Electronic supplementary material

Supplementary Information
