# Supplementary material for: Spatial frequency sensitivity in macaque midbrain
Source: Nat Commun. 2018 Jul 20;9:2852. doi: 10.1038/s41467-018-05302-5 (PMC6054627; doi:10.1038/s41467-018-05302-5)
Supplement: Supplementary file 1 — Supplementary Information [file 41467_2018_5302_MOESM1_ESM.pdf]

Spatial frequency sensitivity in macaque midbrain

Chen et al.

Spatial frequency sensitivity in macaque midbrain

Chih-Yang Chen[1,2,3], Lukas Sonnenberg[4], Simone Weller[4], Thede Witschel[4], and Ziad M. Hafed[1,3*]

1. Werner Reichardt Centre for Integrative Neuroscience, Tuebingen University, Tuebingen, BW, 72076, Germany
2. Graduate School of Neural and Behavioural Sciences, International Max Planck Research School, Tuebingen University, Tuebingen, BW, 72074, Germany
3. Hertie Institute for Clinical Brain Research, Tuebingen University, Tuebingen, BW, 72076, Germany
4. Master's Program for Neurobiology, Tuebingen University, Tuebingen, BW, 72076, Germany

Supplementary Figures

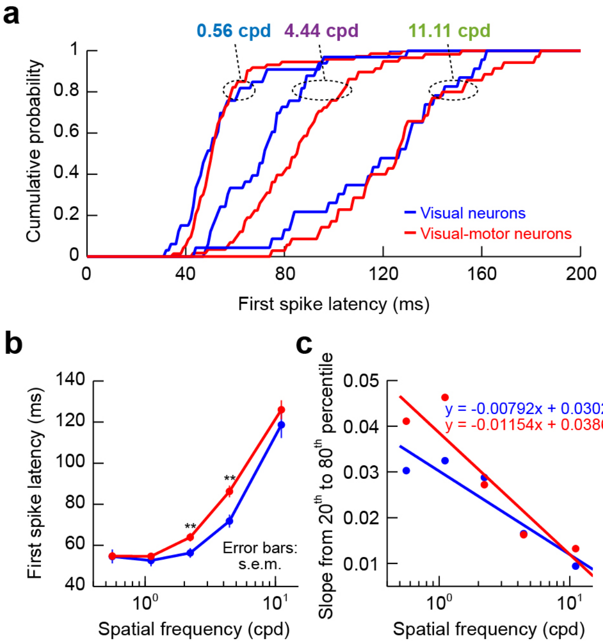

Supplementary Figure 1 Response latencies of visual and visual-motor macaque SC neurons. (**a**) Cumulative histograms of first-spike latency as in Fig. 4a, but after separating neurons as either being purely visual (blue) or visual-motor (red) (i.e. from either superficial or intermediate SC layers, respectively). To reduce clutter, only 3 spatial frequencies are shown. As can be seen, the dependence of response latency on spatial frequency was similar whether neurons were purely visual or visual-motor, but visual neurons tended to exhibit slightly shorter latencies, especially at 4.44 cpd. (**b**) Mean response latencies across neurons as a function of spatial frequency, as in Fig. 4b, but separating visual and visual-motor neurons. Consistent with **a**, response latency increased with increasing spatial frequency for both types of neurons. Also, again consistent with **a**, visual neurons showed earlier responses than visual-motor neurons, especially for intermediate spatial frequencies. Asterisks imply p<0.01 for the comparison between visual and visual-motor neurons at a given spatial frequency (Ranksum test). Error bars denote s.e.m. (**c**) For both visual and visual-motor neurons, response time variability increased with increasing spatial frequency, as in Fig. 4c.

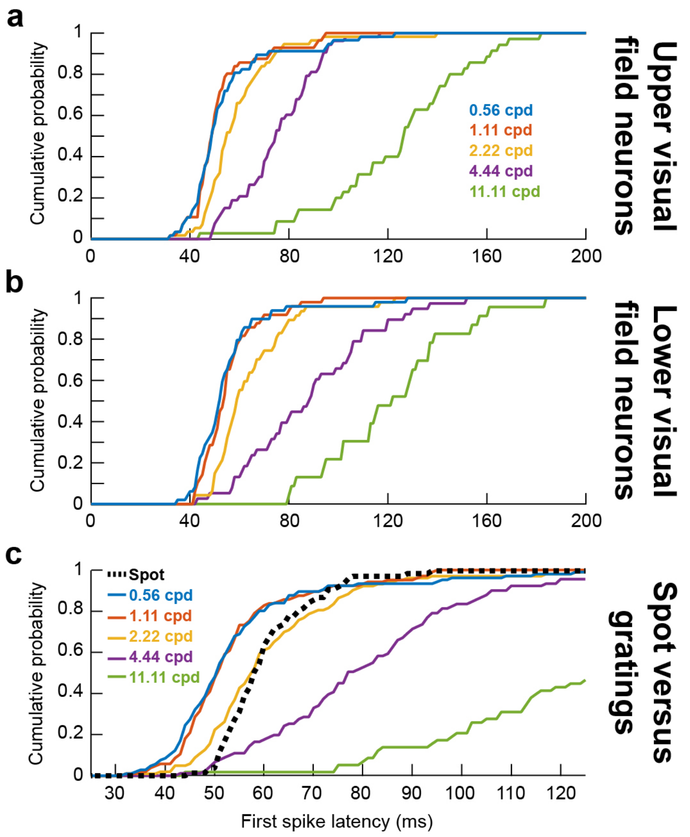

Supplementary Figure 2 Response latencies for upper and lower visual field neurons and for simple spot stimuli. (**a**) Same as Fig. 4a but for neurons only in the upper visual field representation of the SC. (**b**) Same as Fig. 4a but for neurons only in the lower visual field representation of the SC. In both cases, the rank ordering of response latencies as a function of spatial frequency is evident, with an additional observation of upper visual field neurons responding earlier in general than lower visual field neurons (compare the distributions in **a** and **b**), as we showed recently. (**c**) Same as Fig. 4a but now also showing an additional plot (dotted black line) depicting neural response latencies when a small spot of light (like the fixation spot in the main task of our study; Methods) was presented inside the neurons' RF's instead of a sine wave grating. As can be seen, visual response latency for the spot was significantly longer than for the smallest spatial frequencies (0.56 and 1.11 cpd). This might be expected because spots have a broad frequency spectrum that also includes higher spatial frequency components. Also, the spot stimulus was much smaller than gratings presented in peripheral eccentricities.

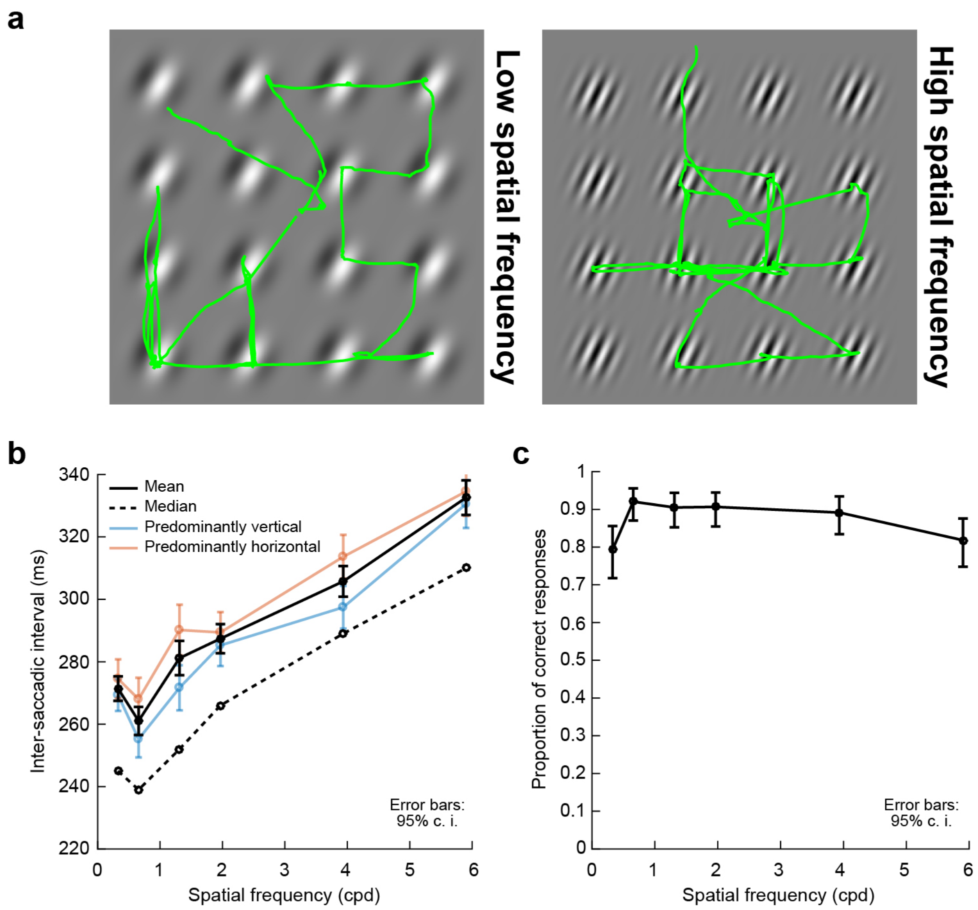

Supplementary Figure 3 Influence of spatial frequency on inter-saccadic intervals during human visual search behavior. (**a**) Example visual search arrays and eye movement scan paths (green) superimposed on them. The left panel shows a search array of targets with a low spatial frequency, and the right panel shows a search array of targets with a higher spatial frequency. In all cases, human subjects searched for an oddball orientation in the array, which was slightly different from the orientation that was present in all other gratings; the task (orientation discrimination) was made difficult enough to require many scanning saccades of the array before the correct oddball stimulus was identified. However, unlike in ref. 1 with masking phenomena, all of our gratings were highly visible (100% contrast; Methods) even at the high spatial frequencies that we tested (contrast at the tested spatial frequencies was well above threshold contrasts of the human contrast sensitivity function). (**b**) Mean (solid black) and median (dashed black) inter-saccadic intervals during target search as a function of the spatial frequency of the targets in the search array. Error bars denote 95% confidence intervals. Inter-saccadic intervals, a correlate of RT in instructed-saccade tasks, progressively increased with higher spatial frequencies, as in our earlier monkey neural and behavioral results. The faint colored lines show results from trials in which the orientations in the stimulus array were either predominantly (within +/- 45 deg) vertical or predominantly horizontal. The increase in inter-saccadic intervals with spatial frequency was equally present for the different orientations. (**c**) Proportion of correct oddball identifications (Methods) as a function of spatial frequency in the target search array. Subjects faithfully searched the array until they could correctly identify the target on the great majority of trials, meaning that the changes in inter-saccadic intervals in **b** were not due to potential speed-accuracy tradeoffs during search. For example, correct target identifications were approximately constant between ~1 and ~4 cpd in this panel (error bars denote 95% confidence intervals), but inter-saccadic interval durations were highly dependent on spatial frequency in the same spatial-frequency range (see **b**). Such ceiling performance is also consistent with evidence[2] that orientation discrimination thresholds do not deteriorate with increasing spatial frequency. Moreover, for all but the very last 1-2 inter-saccadic intervals in **b**, the oddball target was not yet identified by the subjects, so the shorter intervals for low spatial frequencies were not because oddball targets were already identified or recognized. Therefore, even in natural searching gaze behavior with a stimulus that is less prone to visual masking as in ref. 1, the effects of spatial frequencies on inter-saccadic intervals are consistent with a role of the SC *and* other visual brain areas in facilitating and over-representing low spatial frequencies prevalent in natural environments. Error bars denote 95% confidence intervals.

1 White, B. J., Stritzke, M. & Gegenfurtner, K. R. Saccadic facilitation in natural backgrounds. *Curr Biol* **18**, 124-128 (2008).

2 Burr, D. C. & Wijesundra, S.-A. Orientation discrimination depends on spatial frequency. *Vision Res* **31**, 1449-1452 (1991).
